# Uncoupling Protein 2 Deficiency Enhances NLRP3 Inflammasome Activation Following Hyperglycemia-Induced Exacerbation of Cerebral Ischemia and Reperfusion Damage In Vitro and In Vivo

**DOI:** 10.1007/s11064-021-03270-9

**Published:** 2021-03-18

**Authors:** Ting Zhang, Mao-Tao He, Xiao-Peng Zhang, Li Jing, Jian-Zhong Zhang

**Affiliations:** 1 School of Basic Medical Sciences, Ningxia Key Laboratory of Vascular Injury and Repair, Ningxia Medical University, Yinchuan, 750004 Ningxia China; 2 Department of Pathology, General Hospital of Ningxia Medical University, Yinchuan, 750004 Ningxia China

**Keywords:** Uncoupling protein 2, Cerebral ischemia, Hyperglycemia, Reactive oxygen species, Nod-like receptor protein-3

## Abstract

Mitochondrial uncoupling protein 2 (UCP2) deficiency exacerbates brain damage following cerebral ischemia/reperfusion (I/R). The Nod-like receptor protein-3 (NLRP3) inflammasome also plays a vital role in cerebral I/R damage. However, the effect of UCP2 on NLRP3 inflammasome-mediated hyperglycemia and I/R damage is not clear. In the present study, UCP2-knockout (UCP2^−/−^) and wild-type (WT) mice were used to establish a model of middle cerebral artery occlusion (MCAO) and reperfusion under normo- and hyperglycemic conditions. HT22 cells were established as a model of oxygen–glucose deprivation and reoxygenation (OGD/R) with high glucose to mimic hyperglycemia and I/R in vitro. HT22 cells were treated with/without different concentrations of the UCP2-specific inhibitor genipin for different periods of time. The results showed that UCP2 deficiency significantly increased histopathological changes and apoptosis after cerebral I/R damage in hyperglycemic mice. Moreover, UCP2 deficiency enhanced NLRP3 inflammasome activation in neurons when cerebral I/R damage was exacerbated by hyperglycemia. Furthermore, UCP2 deficiency enhanced NLRP3 inflammasome activation and reactive oxygen species (ROS) production in HT22 cells under OGD/R and high-glucose conditions. UCP2 deficiency aggravated hyperglycemia-induced exacerbation of cerebral I/R damage. UCP2 deficiency also enhanced NLRP3 inflammasome activation and ROS production in neurons in vitro and in vivo. These findings suggest that UCP2 deficiency enhances NLRP3 inflammasome activation following hyperglycemia-induced exacerbation of cerebral I/R damage in vitro and in vivo. UCP2 may be a potential therapeutic target for hyperglycemia-induced exacerbation of cerebral I/R damage.

## Introduction

Ischemic stroke is identified as one of the major causes of disability and mortality worldwide. Hyperglycemia is an important independent risk factor for ischemic stroke and induces the occurrence and development of cerebral I/R damage [1, 2]. Previous studies have shown that the exacerbation of ROS production, promotion of neuroinflammation, and extensive programmed cell death are possible mechanisms associated with hyperglycemia-exacerbated cerebral I/R damage. Recent evidence suggests that inflammation is a major factor in cerebral I/R damage due to the inflammatory response and the accumulation of inflammatory cells [3, 4]. However, the molecular mechanism by which hyperglycemia exacerbates ischemic brain injury is still unclear. To date, no effective drugs have been found to treat hyperglycemia-exacerbated ischemic brain injury. Thus, research on neuroprotection and targeted inflammation is very important in preventing and treating hyperglycemia-exacerbated cerebral I/R damage.

UCP2 is the inner membrane protein of mitochondria. It has been proposed that UCP2 regulates the mitochondrial potential and reactive oxygen species (ROS) production [5]. To date, several studies have found that UCP2 plays vital roles in cerebral I/R damage. UCP2 deficiency may enhance brain I/R damage by upregulating the protein levels of inflammatory cytokines and inhibiting antioxidants [6]. The neuroprotective effects of UCP2 are likely related to the regulation of ROS and neuroinflammation. However, the effect of UCP2 on the NLRP3 inflammasome in hyperglycemic and ischemic damage is not clear.

The inflammasome plays an important role in neuroinflammation. The NLRP3 inflammasome is a multiprotein complex that comprises NLRP3, ASC and caspase 1. NLRP3 inflammasome activation leads to NLRP3 recruitment of ASC and pro-caspase 1, which causes caspase-1 activation and the release of caspase activation-dependent cytokines, including IL-1β and IL-18. NLRP3 inflammasome activation may trigger or exacerbate neuroinflammation, thereby promoting cerebral ischemia injury [7, 8].

In this study, we hypothesized that UCP2 deficiency enhances NLRP3 inflammasome activation after hyperglycemia-exacerbated cerebral I/R damage. Thus, we used UCP2-knockout (UCP2^−/−^) and wild-type (WT) mice to establish a model of middle cerebral artery occlusion (MCAO) and reperfusion under normo- and hyperglycemic conditions in vivo. HT22 cells were established as a model of oxygen–glucose deprivation and reoxygenation (OGD/R) with high glucose to mimic hyperglycemia and I/R in vitro. HT22 cells were treated with/without different concentrations of the UCP2-specific inhibitor genipin for different times. We evaluated the effects of UCP2 deficiency and the activation of the NLRP3 inflammasome in hyperglycemia-induced exacerbation of cerebral ischemic injury.

## Materials and Methods

### Materials

A TUNEL assay kit (#11684817910) was purchased from Roche (Mannheim, Germany). Streptozotocin (STZ) was purchased from Sigma. An ROS assay kit (#S0033) was obtained from Beyotime (Shanghai, China). NLRP3 (ab214185), NeuN (ab104224) and IL-1β (ab9722) antibodies were purchased from Abcam (Cambridge, UK). ASC (D2W8U) and TXNIP (D5F3E) antibodies were obtained from Cell Signaling Technology (MA, USA). Caspase 1 (AF5418) and cleaved caspase-1 (AF4005) antibodies were purchased from Affinity.β-Actin and GAPDH antibodies were purchased from Bioss (Beijing, China). Genipin (Sigma-Aldrich, MO, USA) was diluted to appropriate concentrations in cell culture medium. ELISA kits were purchased from Abcam.

### Animals and Groups

Breeding pairs of UCP2^−/−^ mice on a C57BL/6 background were originally obtained from the Jackson Laboratory (USA). UCP2^−/−^ mice were fed and bred in sterile animal housing, and age-matched male mice were used for the experiments. Male wild-type (WT) C57BL/6 mice (6–8 weeks old) were obtained from Vital River Laboratory (Beijing, China). All experimental protocols and procedures were approved by the Institutional Animal Care and Use Committee of Ningxia Medical University and the ethical review committee of Ningxia Medical University (approval document no. 2017–040). Animal experimental procedures were performed according to the NIH Guide for Care and Use of Laboratory Animals.

In the present study, a total of 54 male WT mice and 58 UCP2^−/−^ mice were used. UCP2^−/−^ and WT mice were randomly divided into the sham (sham-operated) group, NG + I/R (normoglycemic + 1 h MCAO and 24 h reperfusion) group, and HG + I/R (hyperglycemic + 1 h MCAO and 24 h reperfusion) group. Hyperglycemia was induced in male mice by streptozotocin (STZ) (120 mg/kg, body weight, i.p.). The blood glucose level was measured with a blood glucose meter 72 h after STZ injection. According to our previous experience, hyperglycemia was designated as a fasting blood glucose level of more than 16.8 mmol/L [9].

### Middle Cerebral Artery Occlusion Model

Mice were anesthetized with 2–3% isoflurane during induction, and anesthesia was maintained with 1.5–1.8% isoflurane during the operation. The right middle cerebral artery occlusion (MCAO) model was established using the filament method. Briefly, the right common carotid artery (CCA), internal carotid artery (ICA), and external carotid artery (ECA) were first separated. Second, a small incision was made in the CCA, and a filament (Doccol Corporation, USA) was inserted into the ICA to block the MCA. Finally, after 1 h of occlusion, animals were anesthetized again, the filament was removed, and reperfusion was performed for 24 h. The mice in the sham group were subjected to the same surgical procedures without blocking the MCA. During the surgery period, the body temperature of the animal was monitored and maintained at 37  ±  0.5 °C with an animal body temperature maintenance instrument (RWD Life Science, Shenzhen, China). The average body temperature of animal during the operation was quantified. After operation, the animal was put into animal intensive care units (Lyon Technologies, Los Angeles, USA) for recovery. The neurological deficit was scored by Zea-Longa’s scale [10]: (0) no neurological deficits; (1) failure to fully extend left paw; (2) circling to the left; (3) falling to the left; (4) unable to walk spontaneously and exhibiting depressed levels of consciousness. The mice were subjected to a neurological examination immediately after the animals recovered from anesthesia to judge the successful induction of MCAO model. The animal with scores of two and above was selected as the successful MCAO model [11]. Four animals with Zea-Longa’s score less than two were excluded from the study. The animals were subjected to a neurological examination again after 24 h reperfusion to compare the neurological deficit between the experiential groups. All animals were coded with a number and the people who further process the measurements and analysis were blinded to the experimental conditions.

### Hematoxylin–Eosin (HE) Staining

First, the mouse brains were made into paraffin blocks of brain tissue. Then, the brain tissue block was sliced into 4 μm sections using a microtome. The sections were deparaffinized and rehydrated in a gradient of xylene and ethanol (xylene I for 10 min, xylene II for 10 min, 100% ethanol I for 10 min, 100% ethanol II for 10 min, 90% ethanol for 5 min, 80% ethanol for 5 min, 70% ethanol for 5 min, 60% ethanol for 5 min, 50% ethanol for 5 min, and ddH_2_O for 5 min). Then, the sections were stained with hematoxylin for 4 min. Next, the sections were stained with eosin for 1 min. Finally, the sections were dehydrated and gently covered with coverslip slides. Images were acquired under a high-power field (400 X) with a microscope, and the percentage of karyopyknotic cells was calculated. The cortical area in five brain slices per mouse was quantified.

### TdT-Mediated dUTP Nick-end Terminal dUTP Nick-End Labeling (TUNEL) Assay

For the detection of apoptosis, brain sections were stained with a TUNEL kit (Roche, #11684795910) according to the manufacturer’s protocols. Briefly, the sections were deparaffinized and rehydrated in a gradient of ethanol. Then, proteinase K working solution was used to digest the tissues for 30 min at 37 °C. After rinsing, the TUNEL reaction mixture was added to the sections for 45 min at 37 °C. Finally, DAPI was applied to the sections, and the sections were covered with a coverslip. TUNEL-positive cells were observed with a fluorescence microscope, and the percentage of TUNEL-positive cells was calculated.

### Cell Culture and Groups

The mouse hippocampal neuronal cell line HT22 was originally obtained from ATCC (Rockville, MD, USA). HT22 cells were maintained in Dulbecco’s modified Eagle’s medium (DMEM) (HyClone laboratories, Waltham, MA) supplemented with 1% penicillin/streptomycin (HyClone) and 10% fetal bovine serum (FBS, HyClone). The cells were cultured in an incubator at 37 °C with 90% relative humidity and 5% CO_2_. The cell medium was replaced per 24 h.

The high glucose, genipin and OGD/R groups were as follows: NG group (control treatment of HT22 cells); HG group (high glucose treatment of HT22 cells); NG+OGD/R group (OGD/R treatment of HT22 cells); HG + OGD/R group (OGD/R and high glucose treatment of HT22 cells); NG + OGD/R + GE group (OGD/R and genipin treatment of HT22 cells); and HG + OGD/R + GE group (OGD/R, high glucose, and genipin treatment of HT22 cells). According to previous studies, the selected high glucose concentration was 50 mM. Glucose was dissolved in PBS to prepare a stock solution and added to the medium at a final concentration of 50 mM. The concentration of glucose in the DMEM that was used was 25 mM. Cells cultured in culture medium containing 25 mM glucose were used as normoglycemic controls.

### Oxygen–Glucose Deprivation/Reoxygenation Model and Drug Treatment

First, the cell culture medium was changed to glucose-free medium, and then the cells were placed in a hypoxic incubator (MIC-101, Billups-Rothenberg Inc) containing 95% N_2_ and 5% CO_2_ at 37 °C for 1 h. Finally, the glucose-free medium was replaced with normal medium and reoxygenated for 24 h.

For genipin treatment, HT22 cells were plated on 96-well plates (5000 cells/well) 24 h before the treatments. After adherence, the cells were treated for 24 h with different concentrations of genipin (25, 50, 75, and 100 μM). At the end of the incubation, cell viability was determined via cell counting kit-8 (CCK-8) (Rockville, MD).

### Cell Viability Assay

In brief, HT22 cells were plated at 5000 cells/well in 96-well plates. The cells were exposed to different treatments after 24 h of adhesion. After an additional 24 h of incubation, the absorbance was recorded by a SpectraMax reader. The relative intensity of the control group was designated as 100% cell viability, and the corresponding percentages of the experimental groups were calculated relative to the control group.

### ROS Production Assay

HT22 cells were incubated in culture medium containing 20 μmol/L dihydroethidine (DHE) for 30 min in the different groups, washed twice with PBS, and fixed in 4% paraformaldehyde in PBS for 15 min. Intracellular ROS production was assessed with a fluorescence microscope. Images were captured at 400 X magnification, and the intensity was evaluated by IPP6.0 image analysis software.

### Western Blot

Mouse brain tissues or HT22 cells were lysed in RIPA lysis buffer containing protease inhibitor cocktails. The protein concentration was measured using a BCA assay kit. Equal amounts of protein (50 µg/well) were loaded into wells for 10% SDS-PAGE, and the resultant gel was transferred to polyvinylidene fluoride membranes. The membrane was incubated with primary antibodies in blocking buffer at 4 °C overnight. The following primary antibodies were used: NLRP3 (1:300), ASC (1:1000), caspase 1 (1:1000), cleavedcaspase 1 (1:1000), UCP2 (1:500), superoxide dismutase (SOD2) (1:1000), TXNIP (1:1500), IL-1β (1:2000), and pro-IL-1β (1:1000). GAPDH (1:1000) and β-actin (1:5000) were used as internal controls for protein loading. Then, the membrane was incubated with secondary antibodies at room temperature for 1 h. Imaging was performed using a BIO-RAD imaging system, and the ratio of the target protein level to GAPDH or β-actin was calculated.

### Immunofluorescence

The paraffin-embedded brain sections were boiled for antigen retrieval in citrate buffer. Then, the sections were blocked for 20 min with 10% goat serum at room temperature. Next, the sections were incubated with primary antibodies at 4 °C overnight. After being washed three times with PBS, the sections were incubated with secondary antibodies at 37 °C for 1 h, and DAPI was added for 5 min in the dark.

HT22 cells from the different groups were seeded on glass slides. The slides of cells were fixed with 4% paraformaldehyde and permeabilized with 0.2% Triton X-100. The following steps were the same as those described above.

### ELISA

The levels of IL-1β and IL-18 in the cell culture supernatant and serum were measured by mouse IL-1β and IL-18 ELISA kits (Abcam, USA) according to the manufacturer's instructions.

### Statistical Analysis

Each experiment was independently repeated a minimum of three times. Statistical analysis was performed with IBM SPSS Statistics version 20.0. All values are expressed as the mean ± standard deviation (SD) or as a percentage of the control. The datasets were analyzed by ANOVA followed by the LSD post-hoc test. Statistical significance was set at *p* < 0.05.

## Results

### UCP2 Deficiency Exacerbated Cerebral I/R-Induced Histopathological Changes and Apoptosis in Hyperglycemic Mice

**Fig. 1:**
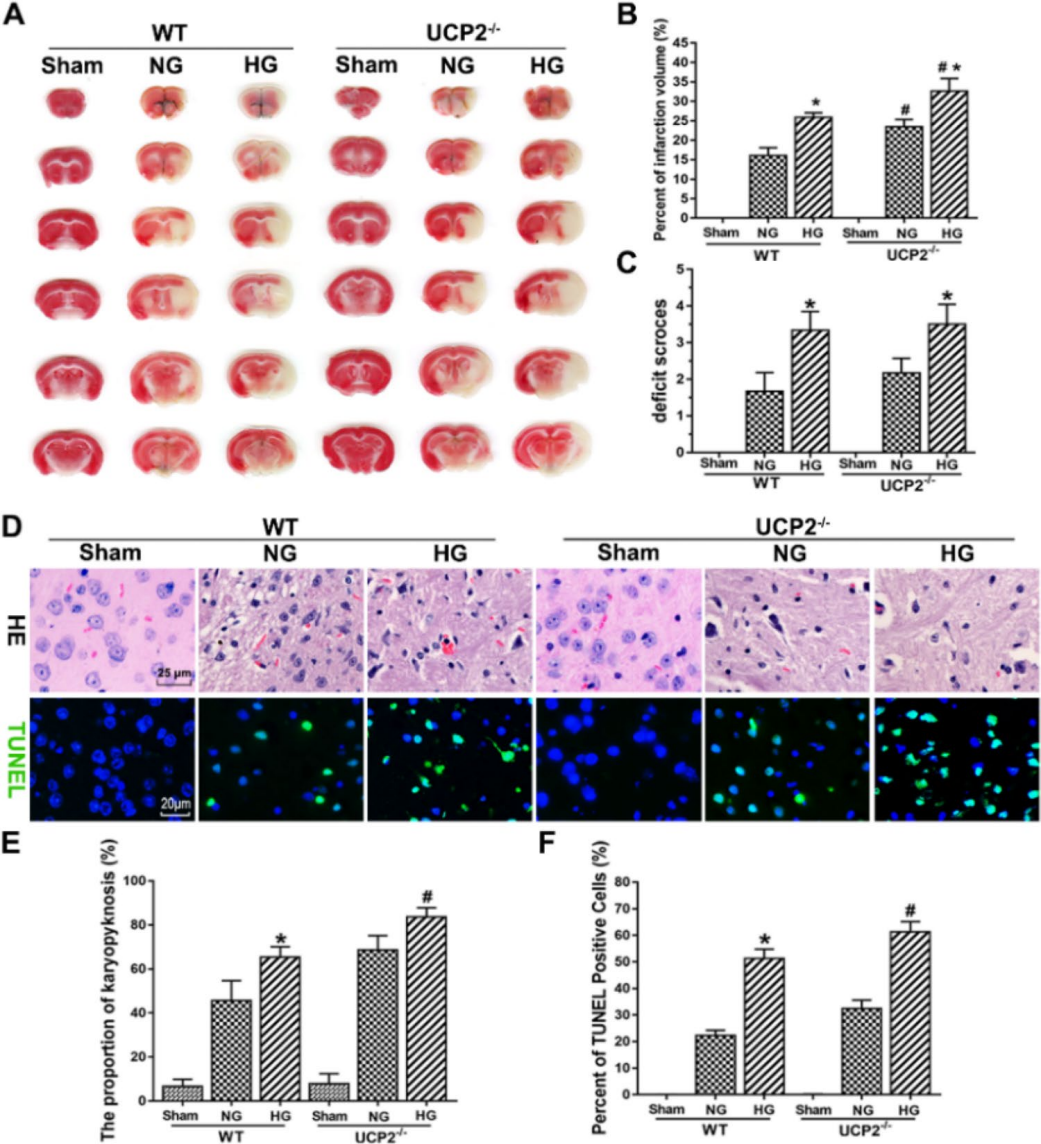
UCP2 deficiency exacerbated I/R-induced histopathological changes and apoptosis in hyperglycemic mice. **a** Representative images of TTC staining in each group. **b** Bar graph summarizing the percent infarction volume in WT and UCP2^−/−^ mice. **c** Assessment of neurological deficits. **d** HE staining and TUNEL staining. **e** Quantitative summary of pyknotic cells. **f** Bar graph summarizing the percentage of TUNEL-positive cells. ^#^*p* < 0.05 vs. the WT group; *p < 0.05 vs. the NG group (n = 6 in each group)

To determine whether UCP2 deficiency exacerbates cerebral I/R damage in hyperglycemic mice, infarct volume was evaluated by TTC staining. Figure 1a and b show that the infarct volume was increased in hyperglycemic mice compared with normoglycemic mice. UCP2 deficiency significantly increased the infarct volume compared with that of WT mice. Neurological deficit scores were significantly increased in hyperglycemic mice compared with normoglycemic mice. As expected, blood glucose levels were significantly higher in hyperglycemia mice than in normoglycemic mice. However, there was no difference in blood glucose levels between WT and UCP2^−/−^ animals (Table 1). During the surgery period, the body temperature of the animal was maintained at 37  ±  0.5 °C with an animal body temperature maintenance instrument and recorded the average body temperature during the operation for quantification. The average body temperature during the operation was not statistically different between UCP2^−/−^ and WT animals under the same glycemic conditions (Table 1). The histopathological outcomes in the cortical area are shown in Fig. 1f. In the sham group, only a few dead cells were observed. The number of dead neurons in the cortical area was slightly increased in WT mice after I/R. UCP2 deficiency further increased the number of dead neurons after cerebral I/R in normoglycemic and hyperglycemic mice. Furthermore, the TUNEL assay revealed that the number of TUNEL-positive cells obviously increased after cerebral I/R damage. Similarly, UCP2 deficiency further increased TUNEL-positive cells after cerebral I/R damage in normoglycemic and hyperglycemic mice (Fig. 1f). These results demonstrated that UCP2 deficiency enhanced histopathological changes and apoptosis after cerebral I/R damage in hyperglycemic mice.

**Table 1:** Blood glucose and body temperature among different groups

Group	WT			UCP2^−/−^		
	Sham	NG	HG	Sham	NG	HG
Blood glucose (mmol/L)	6.89 ± 1.24	6.32 ± 1.46	25.73 ± 3.39*	6.53 ± 1.39	7.15 ± 1.86	24.77 ± 5.68*
Body temperature (℃)	36.79 ± 0.32	36.85 ± 0.20	36.74 ± 0.34	36.81 ± 0.37	36.77 ± 0.24	36.76 ± 0.31

*p < 0.05 vs. the NG group (n = 6 in each group). Body temperature: The average body temperature during the operation

### UCP2 Deficiency Enhanced NLRP3 Inflammasome Activation after Cerebral I/R Damage in Hyperglycemic Mice

**Fig. 2:**
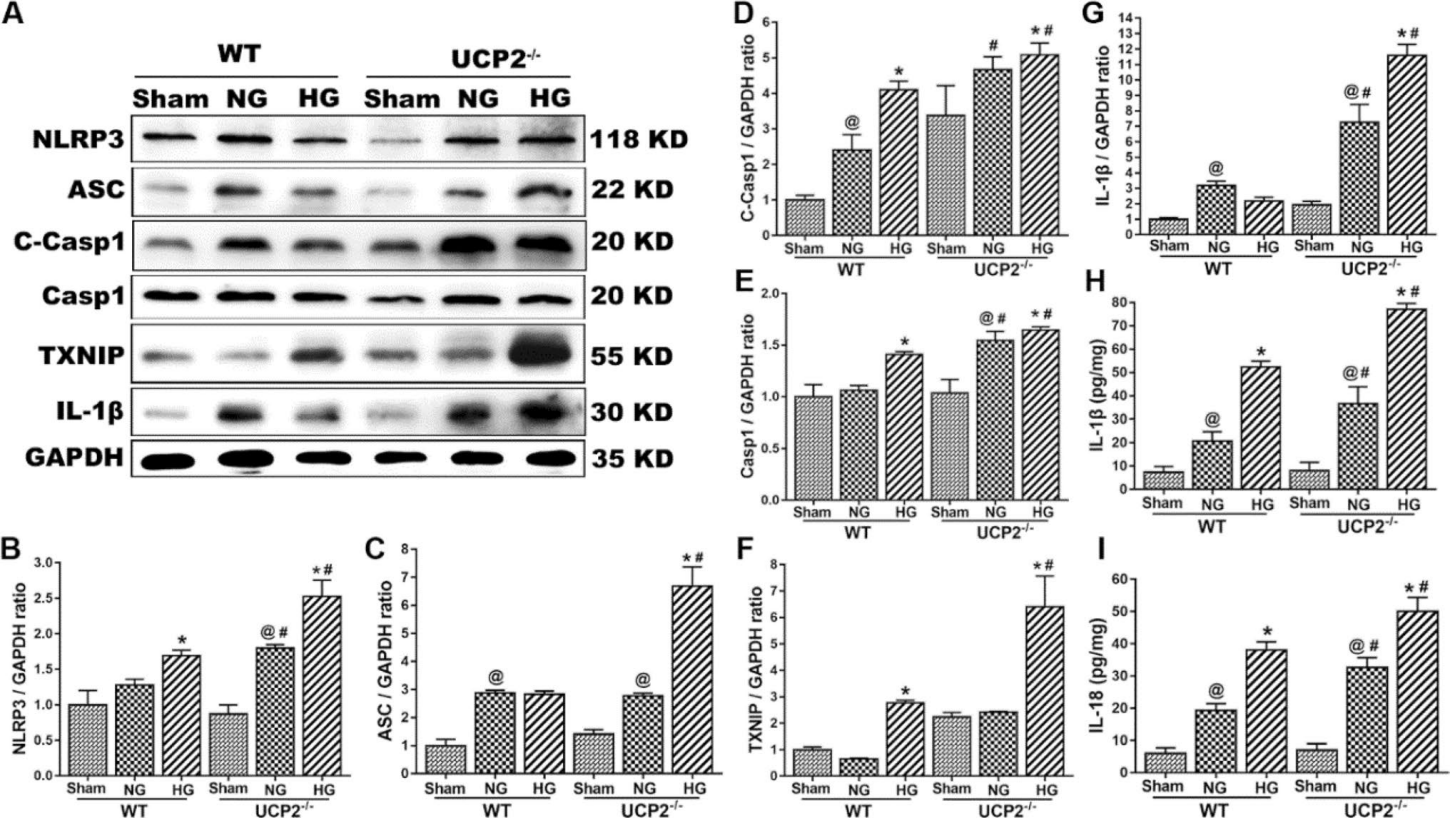
UCP2 deficiency enhanced NLRP3 inflammasome activation after cerebral I/R damage in hyperglycemic mice. **a** The expression of NLRP3, cleaved caspase 1, caspase 1, ASC, TXNIP, and IL-1β in the different groups of UCP2-/- and WT mice. **b**–**g** Semiquantitative analysis of NLRP3, cleaved caspase 1, caspase 1, ASC, TXNIP, and IL-1β protein expression. **h**, **i** The level of IL-1β and IL-18 in serum. ^@^*p* < 0.05 vs. the sham group; ^#^*p* < 0.05 vs. the WT group; ^*^p < 0.05 vs. the NG group (n = 6 in each group)

To determine whether UCP2 deficiency enhances NLRP3 inflammasome activation after cerebral I/R damage, the expression of NLRP3 inflammasome-related proteins was measured by western blotting, and the release of IL-1β and IL-18 was determined by ELISA. The results showed that NLRP3, cleaved caspase 1, and caspase 1 increased after cerebral I/R damage. UCP2 deficiency further increased the expression of NLRP3, cleaved caspase 1, and caspase 1 after cerebral I/R damage in normoglycemic and hyperglycemic mice. Similarly, UCP2 deficiency increased the expression of ASC, TXNIP, and IL-1β after cerebral I/R damage in hyperglycemic mice. The inflammatory cytokines IL-1β and IL-18 were upregulated by UCP2 deficiency after cerebral I/R in hyperglycemic mice (Fig. 2h, i). These results indicated that UCP2 deficiency enhanced NLRP3 inflammasome activation in mice with cerebral I/R damage and hyperglycemia.

### UCP2 Deficiency Promotes NLRP3 Inflammasome Activation in Neurons Under Hyperglycemia-Exacerbated Cerebral I/R Conditions

**Fig. 3:**
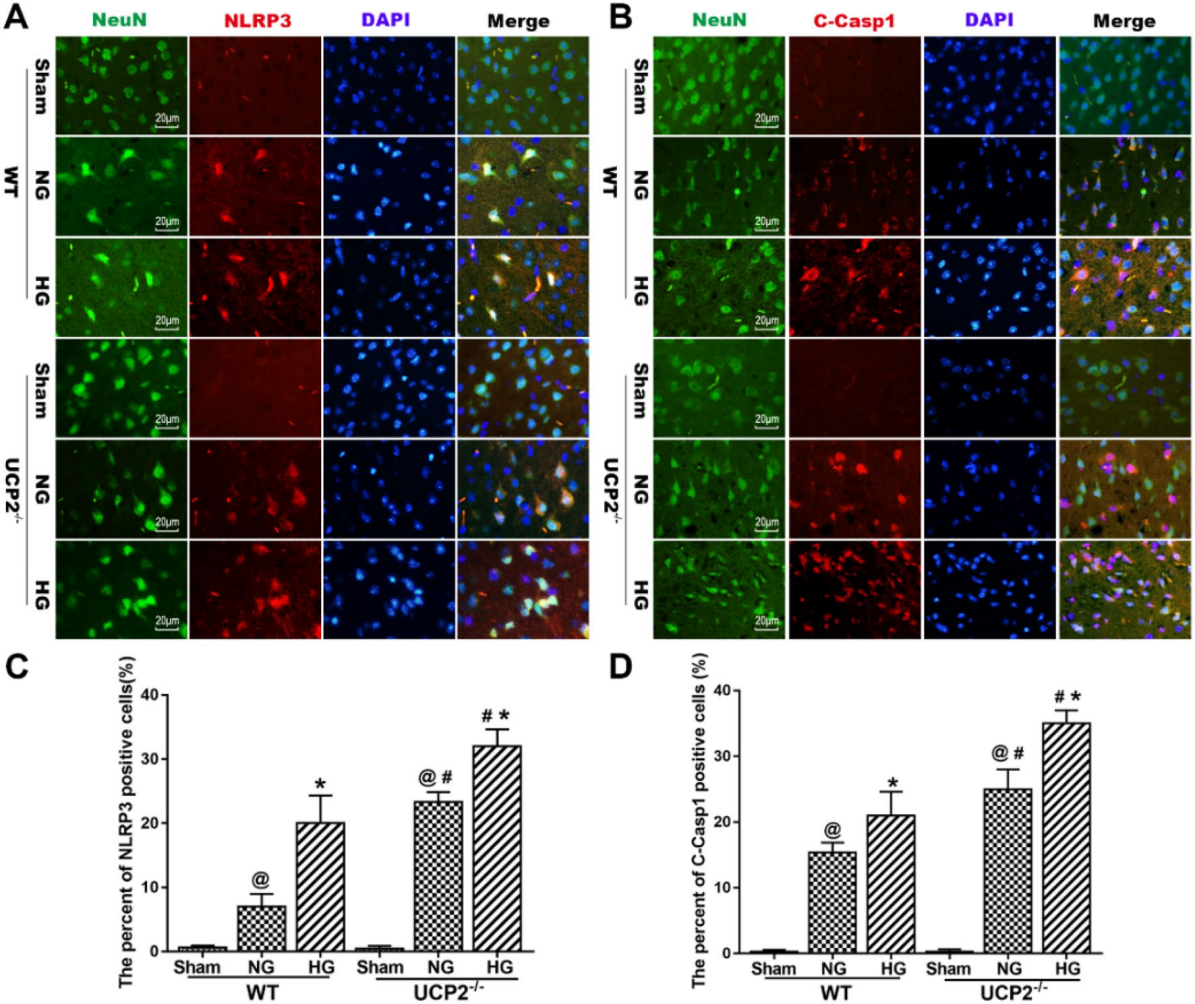
UCP2 deficiency promotes NLRP3 inflammasome activation in neurons under hyperglycemia-exacerbated cerebral I/R conditions. **a** Representative images showing double immunofluorescence staining for NLRP3 (red) and NeuN (green) in the cortical penumbra area. **b** Representative images of double immunofluorescence staining for cleaved caspase 1 (red) and NeuN (green) in the cortical penumbra area. **c** The percentage of NLRP3-positive cells in the cortical penumbra area. **d** The percentage of cleaved caspase 1-positive cells (%) in the cortical penumbra area. ^@^*p* < 0.05 vs. the sham group; ^#^*p* < 0.05 vs. the WT group; *p < 0.05 vs. the NG group (n = 6 in each group)

To further confirm that UCP2 deficiency promotes NLRP3 inflammasome activation in the cortical penumbra area, we used double immunostaining of NLRP3 and cleaved caspase 1 with NeuN (a neuronal marker) in UCP2^−/−^ mouse brain sections after I/R damage. Double labeling of NLRP3 and cleaved caspase 1 with the neuronal marker NeuN (Fig. 3a and b) in brain sections revealed that NLRP3 and cleaved caspase 1 colocalized with NeuN-positive neurons, suggesting that NLRP3 inflammasome activation occurred in neurons after cerebral I/R damage. Immunofluorescence analysis showed that the percentages of NLRP3- and cleaved caspase-1-positive neurons increased after cerebral I/R damage in normoglycemic and hyperglycemic mice. UCP2 deficiency further increased the expression of NLRP3 and cleaved caspase 1 in neurons in the context of hyperglycemia-exacerbated cerebral I/R damage. These results demonstrated that NLRP3 inflammasome activation occurred in neurons in UCP2^−/−^ mice with hyperglycemia-exacerbated cerebral I/R damage. Therefore, this study focused on the effect of UCP2 deficiency on the expression of NLRP3 inflammasome components in neurons in the context of hyperglycemia-exacerbated cerebral I/R damage.

### UCP2 Deficiency Enhanced HT22 Cell Damage Under Oxygen-Glucose Deprivation and Reoxygenation and High Glucose Conditions

**Fig. 4:**
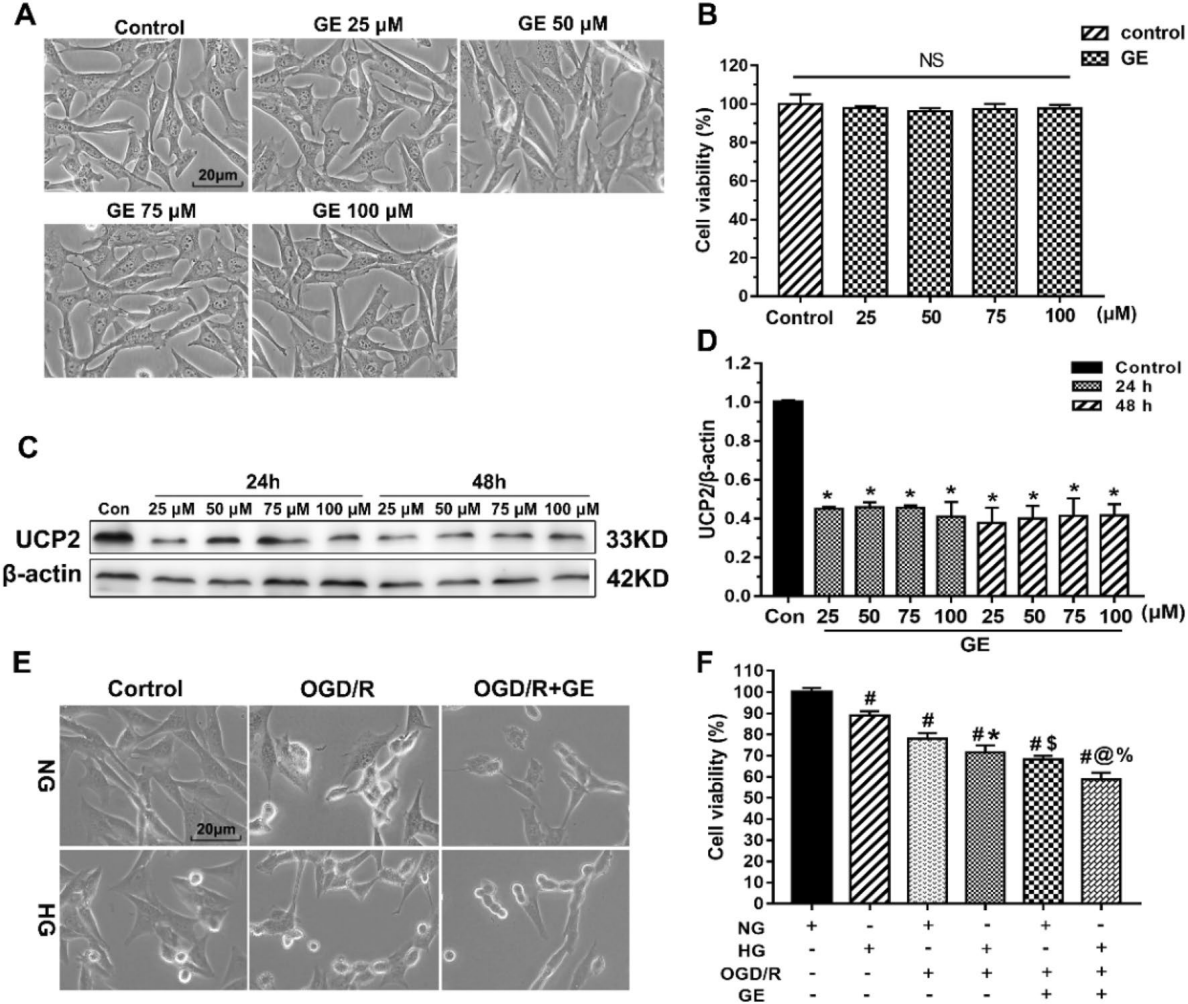
UCP2 deficiency enhanced HT22 cell damage under OGD/R and high glucose conditions. **a** Light microscopy images showing morphological changes in HT22 cells treated with different concentrations of genipin (25 μM, 50 μM, 75 μM, and 100 μM). **b** Cell viability in HT22 cells at different concentrations was determined by CCK-8 assays. **c**, **d** The expression of UCP2 in the different groups at the indicated times was measured by western blotting. *p < 0.05 vs. the Control group. **e** Light microscopic images showing morphological changes in HT22 cells after different treatments. Scale bar = 20 μm. **f** Cell viability analysis of HT22 cells after different treatments. The experiments were repeated three times in triplicate for each condition. ^#^*p* < 0.05 vs. Control; *p < 0.05 vs. HG; ^$^*p* < 0.05 vs. NG + OGD/R; ^@^*p* < 0.05 vs. HG + OGD/R; ^%^*p* < 0.05 vs. NG + OGD/R + GE

Because the NLRP3 inflammasome is expressed in neurons, we used hippocampal HT22 neurons to explore whether UCP2 deficiency affects the NLRP3 inflammasome activation pathway. Genipin is a specific UCP2 inhibitor that was used to explore the role of UCP2 in HT22 cells. To determine the effect of genipin on the expression of UCP2 and cell viability, we treated HT22 neuronal cells with genipin. First, a dose–response experiment was performed on HT22 cells treated with genipin for 24 and 48 h. Figure 4a, b shows that cell viability was not changed in response to genipin (25–100 μM) (*p* > 0.05). Then, the expression of UCP2 was measured by western blotting after genipin treatment. Figure 4c, d shows that the protein expression of UCP2 significantly decreased after 25 μM genipin treatment for 24 h. Increasing the drug concentration and treatment time did not affect the expression of UCP2. Therefore, we chose 25 μM genipin treatment for 24 h for subsequent experiments.

To further assess the influence of UCP2 deficiency on HT22 cells exposed to OGD/R and high glucose conditions, we assessed cell viability with a CCK-8 kit. Figure 4e shows representative images of HT22 cell morphology under a light microscope. OGD/R resulted in cell shrinkage and nuclear condensation, suggesting cell damage. After the addition of genipin to inhibit UCP2 expression, cell damage worsened. The CCK-8 results (Fig. 4f) showed that NG + OGD/R decreased cell viability to 78% and that OGD/R + HG further decreased cell viability to 71%. After the addition of genipin to inhibit UCP2 expression, NG + OGD/R reduced cell viability to 68%, and HG + OGD/R further reduced cell viability to 58%. These results indicated that UCP2 deficiency enhanced HT22 cell damage under OGD/R and high glucose conditions.

### UCP2 Deficiency Enhanced NLRP3 Inflammasome Activation in HT22 Cells Under OGD/R and High Glucose Conditions

**Fig. 5:**
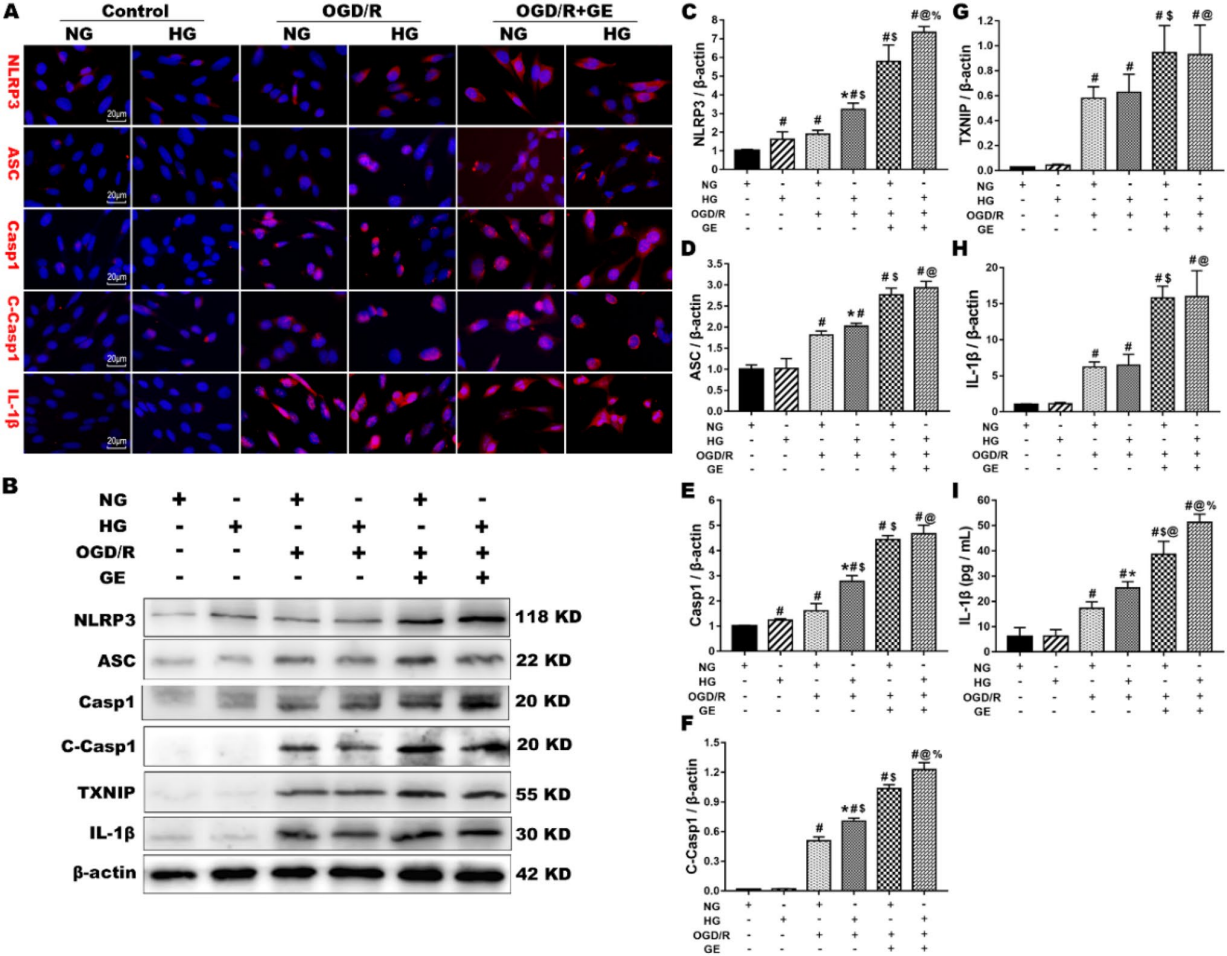
UCP2 deficiency enhanced NLRP3 inflammasome activation in HT22 cells under OGD/R and high glucose conditions. **a** Double immunostaining of NLRP3, ASC, Caspase 1, Cleaved Caspase 1, and IL-1β (red) with DAPI (blue)) was performed in the different groups. **b** Representative western blots showing NLRP3, ASC, Caspase 1, Cleaved Caspase 1, TXNIP, and IL-1β in the different groups. **c**–**h** Quantification of the average NLRP3, ASC, Caspase 1, Cleaved Caspase 1, TXNIP, and IL-1β expression normalized to β-actin. **i** The level of IL-1β in HT22 cell culture medium. The experiments were repeated three times in triplicate for each condition. ^#^*p* < 0.05 vs. Control; *p < 0.05 vs. HG; ^$^*p* < 0.05 vs. NG + OGD/R; ^@^*p* < 0.05 vs. HG + OGD/R; ^%^*p* < 0.05 vs. NG + OGD/R + GE

To investigate the role of UCP2 deficiency on NLRP3 inflammasome activation in HT22 cells under OGD/R and high glucose conditions, we measured the expression of NLRP3 inflammasome proteins, including NLRP3, ASC, cleaved caspase 1, caspase 1, TXNIP, and IL-1β, using western blotting and immunofluorescence. Western blotting showed that OGD/R increased the protein levels of NLRP3, ASC, cleaved caspase 1, caspase 1, TXNIP, and IL-1β. Concurrent treatment with genipin further increased the expression of NLRP3, ASC, cleaved caspase 1, caspase 1, TXNIP, and IL-1β in HT22 cells under normal and high glucose conditions. These results suggest that OGD/R activates the NLRP3 inflammasome in HT22 cells. After genipin was added to inhibit UCP2 expression, the NLRP3 inflammasome was further activated. Immunofluorescence analysis confirmed the changes in NLRP3, ASC, cleaved caspase 1, caspase 1 and IL-1β (Fig. 5a). The results showed that almost no neurons were positive for NLRP3, ASC, caspase 1, cleaved caspase 1 or IL-1β in the normal control cells. OGD/R increased the immunoreactivity of NLRP3, ASC, cleaved caspase 1, caspase 1 and IL-1β in the cytoplasm under normal and high glucose conditions. Genipin treatment significantly increased the number of NLRP3-, ASC-, caspase 1-, cleaved caspase 1-, and IL-1β-positive cells in the NG + OGD/R and HG + OGD/R groups. Similarly, the ELISA results showed that OGD/R caused significant increases in IL-1β, and genipin treatment further increased the level of IL-1β in HT22 cell culture medium under normal and high glucose conditions. These results indicated that UCP2 deficiency enhanced NLRP3 inflammasome activation in HT22 cells under OGD/R and high glucose conditions.

### UCP2 Deficiency Enhanced the Production of ROS in HT22 Cells under OGD/R and High Glucose Conditions

**Fig. 6:**
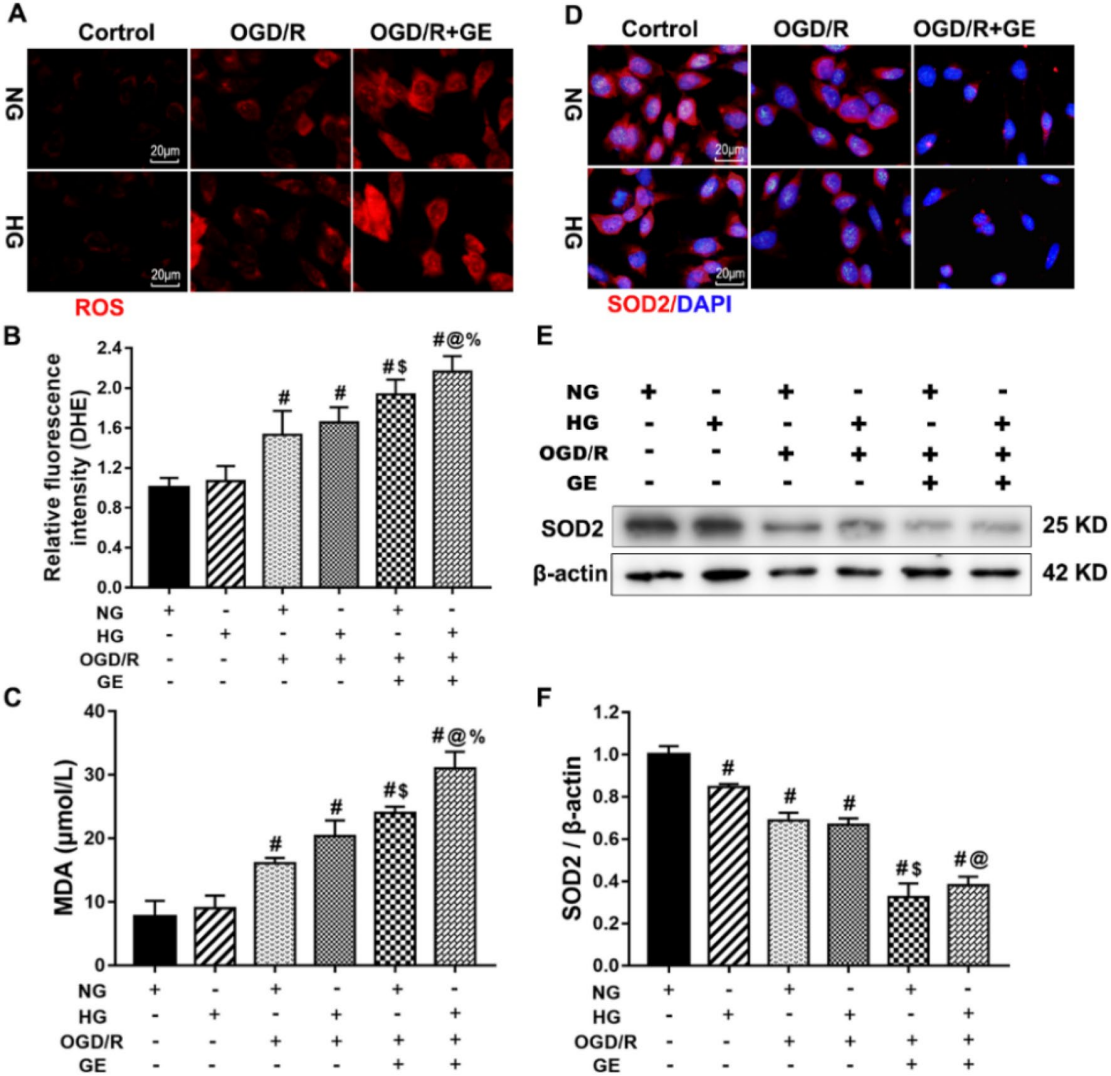
UCP2 deficiency enhanced the production of ROS in HT22 cells under OGD/R and high glucose conditions. **a** DHE fluorescence. ROS were detected by the DHE probe (Red). **b** The presence of ROS was determined using DHE. **c** The MDA level in the different groups of HT22 cells. **d** SOD2 fluorescence. SOD (green color) was detected using antibodies. DAPI (blue color) labeled the nuclei. **e** Representative western blots showing SOD2 expression. **f** Quantification of the average SOD2 expression normalized to β-actin. The experiments were repeated three times in triplicate for each condition. ^#^*p* < 0.05 vs. Control; *p < 0.05 vs. HG; ^$^*p* < 0.05 vs. NG + OGD/R; ^@^*p* < 0.05 vs. HG + OGD/R; ^%^*p* < 0.05 vs. NG + OGD/R + GE

Excess ROS production causes different forms of DNA damage, resulting in cell death. We measured ROS production using the DHE probe. As shown in Fig. 6a, b, and c, OGD/R and HG + OGD/R caused significant increases in ROS and MDA production compared with those of the control group (*p* < 0.01). Simultaneous treatment with genipin resulted in obvious increases in OGD/R-induced ROS and MDA production under normal or high glucose conditions (*p* < 0.01). Manganese SOD (SOD2/MnSOD) is a mitochondria-resident enzyme that functions as the first line in mitochondrial antioxidant defense. We measured the protein levels of SOD2 in the different groups by immunofluorescence and western blotting. The immunofluorescence results showed abundant staining of SOD2-positive neurons among normal HT22 cells. OGD/R obviously reduced the number of SOD2-positive cells. Concurrent treatment with genipin further decreased the immunoreactivity of SOD2 in ODG/R-exposed neurons. Western blot analysis demonstrated that the level of SOD2 protein was lower in the OGD/R group than in the control group, and genipin further decreased the expression of SOD2 compared with that in the OGD/R group under normal or high glucose conditions. These results suggested that UCP2 deficiency enhanced ROS production in HT22 cells under OGD/R and high glucose conditions.

## Discussion

Our study showed that UCP2 deficiency significantly increased histopathological changes and apoptosis after cerebral I/R in normoglycemic and hyperglycemic mice. Furthermore, UCP2 deficiency enhanced NLRP3 inflammasome activation in neurons in the context of hyperglycemia-exacerbated cerebral I/R damage. Moreover, UCP2 deficiency enhanced NLRP3 inflammasome activation and ROS production in HT22 cells under OGD/R and high glucose conditions. These findings suggest that UCP2 deficiency enhances NLRP3 inflammasome activation and ROS production in neurons in the context of hyperglycemia-exacerbated cerebral I/R damage in vivo and in vitro.

UCP2 plays important roles in the process of cerebral I/R damage. The main function of UCP2 may be to stabilize the inner mitochondrial membrane potential and reduce ROS generation [5]. Previous studies indicated that UCP2 deficiency exacerbates infarct volume and mitochondrial dysfunction in normoglycemic and hyperglycemic animals subjected to cerebral I/R damage [11]. UCP2 deficiency upregulates inflammatory cytokines and suppresses antioxidants, and UCP2 overexpression inhibits proinflammatory cytokine expression and activates cell survival factors [12, 13]. The neuroprotective effects of UCP2 are likely related to the regulation of ROS and neuroinflammation. Our data demonstrated that UCP2 deficiency significantly increased brain damage and apoptosis after I/R. The above results demonstrated that UCP2 plays important roles in neuroinflammation after cerebral I/R damage.

Recently, the NLRP3 inflammasome has been shown to serve as a critical contributor to cerebral I/R damage [14]. Previous studies have reported that the NLRP3 inflammasome is involved in neuronal cell death in ischemic stroke [15]. As has been reported, NLRP3 oligomerizes and recruits ASC. ASC can activate pro-Caspase 1 and induce cleavage into its active fragments. Then, cleaved Caspase 1 induces the formation of mature pro-inflammatory cytokines IL-1β and IL-18 [16, 17]. The inflammatory cytokines IL-1β and IL-18 can cause cellular damage through autophagy and pyroptosis in cerebral I/R damage [18]. The present study indicated that the NLRP3 inflammasome was activated after cerebral I/R in both normoglycemic and hyperglycemic mice. Furthermore, UCP2 deficiency promoted NLRP3 inflammasome activation under hyperglycemia-exacerbated cerebral I/R damage. It has been observed that the levels of the NLRP3 inflammasome proteins IL-1β and IL-18 are upregulated in HT22 cells under OGD/R conditions [19]. However, some studies have shown that NLRP3 is expressed in astrocytes, endothelial cells and microglia but not neurons [14, 20, 21]. Moreover, some reports have shown that the NLRP3 inflammasome is activated in microglia but is not detected in astrocytes [22]. Therefore, it is not yet clear whether NLRP3 inflammasomes are specifically expressed and distributed in the brain after I/R. In this study, the immunofluorescence results showed that NLRP3 inflammasome activation occurred in neurons after I/R. These differences may be due to the different ischemia models, the duration of the ischemic insult, and the different interventions. Therefore, this study focused on the effect of UCP2 deficiency on the expression of inflammasomes in neurons in the context of hyperglycemia-exacerbated I/R damage. Similarly, the expression of NLRP3 and IL-1β increased in HT22 cells treated with high glucose and OGD/R relative to those of the control group. Furthermore, UCP2 deficiency enhanced NLRP3 inflammasome activation after hyperglycemia-exacerbated cerebral I/R damage in vivo and in vitro. However, the molecular and cellular mechanisms of NLRP3 activation remain unclear.

Some studies suggest that ROS are critical stimuli that activate the NLRP3 inflammasome [23, 24]. Experimental evidence demonstrates that an increase in ROS concentration following cellular stress leads to TXNIP-mediated recruitment of NLRP3 and subsequent NLRP3 activation [25]. It has been demonstrated that reducing ROS or NLRP3 or inhibiting Caspase-1 abolishes ROS production and the upregulation of IL-1β and IL-18 induced by ATP or LPS [26]. A previous study showed that soluble uric acid induced NLRP3 inflammasome activation by ROS production [27]. Moreover, tPA may stimulate NLRP3 inflammasome activation by generating increased ROS after hyperglycemia-exacerbated I/R damage [28]. In addition, overproduction of IL-1β could promote ROS production, resulting in oxidative damage after ischemia [23, 25, 29]. Overall, these findings indicate the importance of ROS-mediated promotion of NLRP3 inflammasome activation after cerebral I/R damage. However, many aspects of ROS-dependent NLRP3 inflammasome activation remain unknown. In this study, we found that UCP2 deficiency exacerbated NLRP3 inflammasome activation and increased ROS production in neurons after hyperglycemia-exacerbated cerebral I/R damage in vivo and in vitro. Increasing evidence suggests that UCP2 may regulate mitochondrial potential and ROS production in cerebral I/R damage [30, 31]. The vast majority of published studies have reported that overexpression of UCP2 plays vital protective roles in cerebral I/R damage [31, 32]. Studies have confirmed that the upregulation of UCP2 can reduce ROS production in cerebral I/R damage [33]. Our previous study showed that UCP2 deficiency increased ROS production after cerebral I/R damage [34]. Taken together, the present study suggests that UCP2 deficiency enhances NLRP3 inflammasome activation by increasing ROS production in the context of hyperglycemia-exacerbated I/R damage. Hence, UCP2 is likely an upstream mediator of ROS generation and NLRP3 inflammasome activation after hyperglycemia-exacerbated I/R damage. Nevertheless, the inability to knock out UCP2 or express it at high levels in vitro is a limitation of the current study.

In summary, our data suggest that UCP2 deficiency enhances NLRP3 inflammasome activation and ROS production after neurons in hyperglycemia-exacerbated cerebral I/R damage in vivo and in vitro. UCP2 is likely an upstream mediator of ROS generation and NLRP3 inflammasome activation in hyperglycemia-exacerbated I/R damage. Although the underlying pathological mechanisms behind hyperglycemia-exacerbated I/R damage have not been fully investigated, our study provides a possible novel strategy for the treatment of stroke.

## Abbreviations

UCP2: Uncoupling protein 2
UCP2^−^^/^^−^: UCP2 deficiency
I/R: Ischemia/reperfusion
MCAO: Middle cerebral artery occlusion
WT: Wildtype
NG: Normoglycemic
HG: Hyperglycemic
TUNEL: Terminal deoxynucleotidyl transferase mediated dUTP nick‑end labeling
ROS: Reactive oxygen species
STZ: Streptozotocin
OGD/R: Oxygen–glucose deprivation/reoxygenation
GE: Genipin
CCA: Common carotid artery
DHE: Dihydroethidium
NLRP3: NLR family, pyrin domain containing 3
ASC: Apoptosis associated speck-like protein
TXNIP: Thioredoxin-interacting protein
